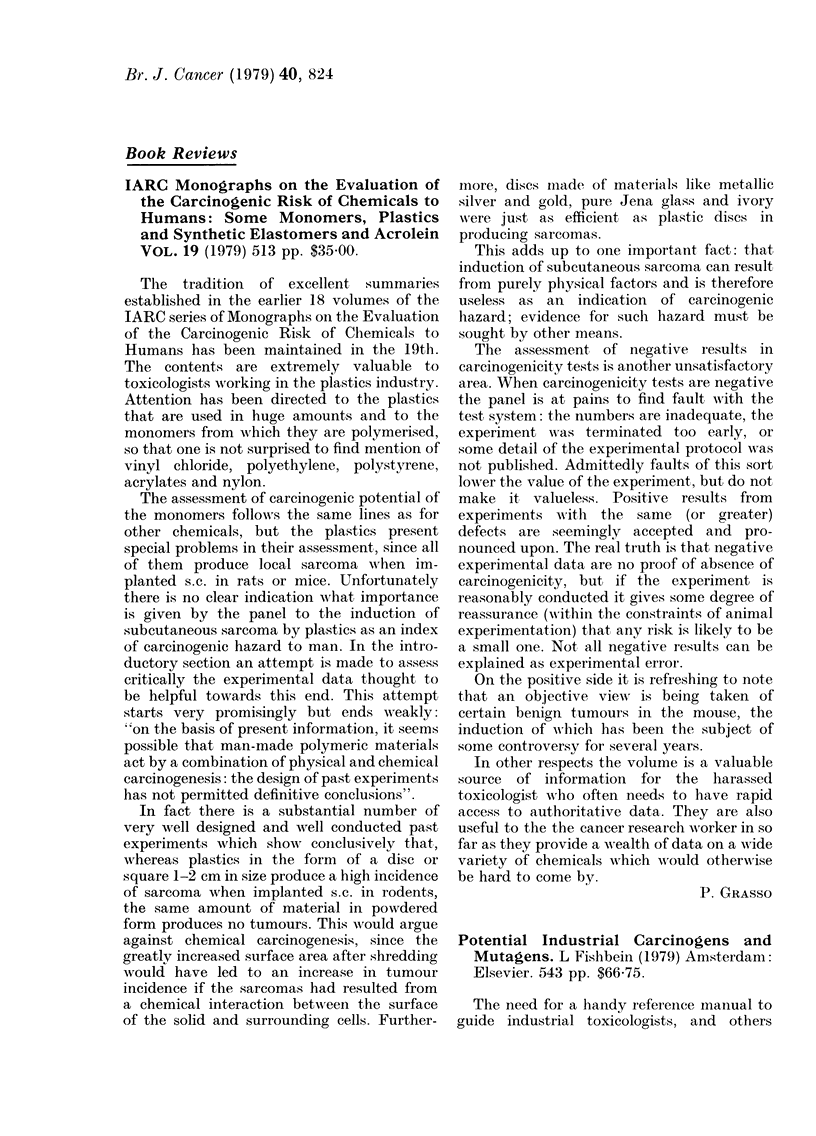# IARC Monographs on the Evaluation of the Carcinogenic Risk of Chemicals to Humans: Some Monomers, Plastics and Synthetic Elastomers and Acrolein Vol. 19

**Published:** 1979-11

**Authors:** P. Grasso


### *Book Reviews*

**IARC Monographs on the Evaluation of the Carcinogenic Risk of Chemicals to Humans: Some Monomers, Plastics and Synthetic Elastomers and Acrolein VOL. 19** (1979) 513 pp. $35·00.

The tradition of excellent summaries established in the earlier 18 volumes of the IARC series of Monographs on the Evaluation of the Carcinogenic Risk of Chemicals to Humans has been maintained in the 19th. The contents are extremely valuable to toxicologists working in the plastics industry. Attention has been directed to the plastics that are used in huge amounts and to the monomers from which they are polymerised, so that one is not surprised to find mention of vinyl chloride, polyethylene, polystyrene, acrylates and nylon.

The assessment of carcinogenic potential of the monomers follows the same lines as for other chemicals, but the plastics present special problems in their assessment, since all of them produce local sarcoma when implanted s.c. in rats or mice. Unfortunately there is no clear indication what importance is given by the panel to the induction of subcutaneous sarcoma by plastics as an index of carcinogenic hazard to man. In the introductory section an attempt is made to assess critically the experimental data thought to be helpful towards this end. This attempt starts very promisingly but ends weakly: "on the basis of present information, it seems possible that man-made polymeric materials act by a combination of physical and chemical carcinogenesis: the design of past experiments has not permitted definitive conclusions".

In fact there is a substantial number of very well designed and well conducted past experiments which show conclusively that, whereas plastics in the form of a disc or square 1–2 cm in size produce a high incidence of sarcoma when implanted s.c. in rodents, the same amount of material in powdered form produces no tumours. This would argue against chemical carcinogenesis, since the greatly increased surface area after shredding would have led to an increase in tumour incidence if the sarcomas had resulted from a chemical interaction between the surface of the solid and surrounding cells. Furthermore, discs made of materials like metallic silver and gold, pure Jena glass and ivory were just as efficient as plastic discs in producing sarcomas.

This adds up to one important fact: that induction of subcutaneous sarcoma can result from purely physical factors and is therefore useless as an indication of carcinogenic hazard; evidence for such hazard must be sought by other means.

The assessment of negative results in carcinogenicity tests is another unsatisfactory area. When carcinogenicity tests are negative the panel is at pains to find fault with the test system: the numbers are inadequate, the experiment was terminated too early, or some detail of the experimental protocol was not published. Admittedly faults of this sort lower the value of the experiment, but do not make it valueless. Positive results from experiments with the same (or greater) defects are seemingly accepted and pronounced upon. The real truth is that negative experimental data are no proof of absence of carcinogenicity, but if the experiment is reasonably conducted it gives some degree of reassurance (within the constraints of animal experimentation) that any risk is likely to be a small one. Not all negative results can be explained as experimental error.

On the positive side it is refreshing to note that an objective view is being taken of certain benign tumours in the mouse, the induction of which has been the subject of some controversy for several years.

In other respects the volume is a valuable source of information for the harassed toxicologist who often needs to have rapid access to authoritative data. They are also useful to the the cancer research worker in so far as they provide a wealth of data on a wide variety of chemicals which would otherwise be hard to come by.

P. GRASSO